# Infrared Comb Spectroscopy of Buffer-Gas-Cooled Molecules: Toward Absolute Frequency Metrology of Cold Acetylene

**DOI:** 10.3390/ijms22010250

**Published:** 2020-12-29

**Authors:** Luigi Santamaria, Valentina Di Sarno, Roberto Aiello, Maurizio De Rosa, Iolanda Ricciardi, Paolo De Natale, Pasquale Maddaloni

**Affiliations:** 1 Agenzia Spaziale Italiana, Contrada Terlecchia, 75100 Matera, Italy; luigi.santamaria@asi.it; 2 Consiglio Nazionale delle Ricerche-Istituto Nazionale di Ottica, Via Campi Flegrei 34, 80078 Pozzuoli, Italy; valentina.disarno@ino.cnr.it (V.D.S.); roberto.aiello@ino.cnr.it (R.A.); maurizio.derosa@ino.cnr.it (M.D.R.); iolanda.ricciardi@ino.cnr.it (I.R.); 3 Istituto Nazionale di Fisica Nucleare, Sez. di Napoli, Complesso Universitario di M.S. Angelo, Via Cintia, 80126 Napoli, Italy; 4 Consiglio Nazionale delle Ricerche-Istituto Nazionale di Ottica, Largo E. Fermi 6, 50125 Firenze, Italy; paolo.denatale@ino.cnr.it; 5 Istituto Nazionale di Fisica Nucleare, Sez. di Firenze, Via G. Sansone 1, 50019 Sesto Fiorentino, Italy

**Keywords:** acetylene, buffer-gas-cooling, infrared frequency combs, precision spectroscopy

## Abstract

We review the recent developments in precision ro-vibrational spectroscopy of buffer-gas-cooled neutral molecules, obtained using infrared frequency combs either as direct probe sources or as ultra-accurate optical rulers. In particular, we show how coherent broadband spectroscopy of complex molecules especially benefits from drastic simplification of the spectra brought about by cooling of internal temperatures. Moreover, cooling the translational motion allows longer light-molecule interaction times and hence reduced transit-time broadening effects, crucial for high-precision spectroscopy on simple molecules. In this respect, we report on the progress of absolute frequency metrology experiments with buffer-gas-cooled molecules, focusing on the advanced technologies that led to record measurements with acetylene. Finally, we briefly discuss the prospects for further improving the ultimate accuracy of the spectroscopic frequency measurement.

## 1. Introduction

Always essential in understanding the molecular structure and dynamics, through the quantum-state-resolved analysis of rotation-vibration spectra [1], high-resolution infrared (IR) spectroscopy has received a new boost in recent decades, first from the technology of optical frequency combs (OFCs) and subsequently from the buffer-gas-cooling (BGC) method [2,3].

Initially invented to link microwave (MW) and optical frequencies for metrological purposes [4,5], OFCs quickly became pervasive in precision spectroscopy, providing straightforward frequency-scale calibration of the spectra with atomic-clock accuracy [6]. In addition, OFCs have opened new avenues in many other fields, encompassing fundamental time metrology, range finding, astronomy, optical sampling, and ultralow-noise microwave synthesis [7]. Their extraordinary impact on science was reflected by the attribution of the Nobel Prize in Physics in 2005 to Theodor W. Hänsch and John L. Hall.

First demonstrated by the group of F. C. De Lucia under the name of collisional cooling [8], BGC represents today the main road for producing low-temperature ground-state molecules in the range of a few Kelvin [9,10], also for the implementation of secondary cooling/trapping strategies towards the achievement of quantum degeneracy [11]. In particular, cooling of rotational and translational temperatures considerably improves the quality of ro-vibrational spectra, which exhibit reduced spectral congestion, enhanced absorption cross sections, and narrow Doppler linewidths or, in Lamb-dip detection, reduced transit-time broadening.

The purpose of this review is to survey the currently available various types of OFCs and BGC sources which can be conveniently combined for carrying out new kinds of precision molecular spectroscopic measurements in the IR domain. The paper is organized as follows. Section 2 discusses the state of the art of IR OFCs, based either on nonlinear optical processes, such as difference frequency generation (DFG) and optical parametric oscillation (OPO), or on direct laser emission like in quantum cascade lasers (QCLs). Section 3 describes the preparation of cold molecular samples for spectroscopic purposes using the most advanced BGC sources. Then, Section 4 describes the most relevant achievements in the field of OFC-based ro-vibrational spectroscopy of buffer-gas-cooled neutral molecules, both in direct frequency comb spectroscopy (DFCS), where the OFC is used as a direct probe source, and in frequency-comb-assisted spectroscopy (FCAS), where the OFC is used as an accurate ruler for absolute metrology. In the latter case, we focus on the first demonstration of Lamb-dip spectroscopy on a buffer-gas-cooled sample, as recently reported by our group. Conclusions are drawn in Section 5, with an emphasis on some significant technological upgrades, addressing the spectroscopic/metrological scheme, which may substantially enhance the accuracy with which center frequencies of ro-vibrational transitions are measured, mainly directed to improved tests of fundamental Physics.

## 2. Infrared Frequency Combs for Molecular Spectroscopy

An OFC is a laser source whose emission spectrum is constituted by a broad set of narrow, evenly spaced and highly coherent optical modes (also called comb teeth), fully controlled through only two parameters: the frequency separation between adjacent modes (mode spacing) and a common offset frequency.

### 2.1. Working Principle and Basic Properties

OFCs were originally obtained from phase-stabilized femtosecond (fs) mode-locked lasers (MLLs), which therefore represent an excellent starting point to illustrate the fundamental comb generation mechanism and the resulting spectral properties [12,13]. In the time domain, a MLL emits a train of ultrashort pulses. The period of the envelope of the pulses is given by $1/f_{rep}=L/v_{g}$, where *L* is the round-trip length of the laser cavity and $v_{g}$ the group velocity of the light. Due to dispersion inside the cavity, the group and the phase velocity are not equal, resulting in a pulse-to-pulse phase shift Δϕ between the carrier and the envelope of the pulses. In the frequency domain, the associated spectrum consists of a series of discrete, harmonically related sharp lines at frequencies

(1) $$f_{n}=f_{ceo}+n\;f_{rep}\;.$$

In the above equation, the mode spacing coincides with the laser repetition rate, $f_{rep}$, and the ruler offset is given by $f_{ceo}=\Delta \phi \cdot f_{rep}/2\pi$, so-called carrier-envelope offset frequency. Ultimately, by virtue of the large integer number *n* (up to a few million), a bridge is established between optical frequencies ($f_{n}$) and the radio-frequency (RF) domain, where both $f_{rep}$ and $f_{ceo}$ fall. To give an idea, since MLLs used as OFCs have typical optical cavity lengths between 30 cm and 3 m, $f_{rep}$ ranges from 1 GHz to 100 MHz, correspondingly. By definition, $f_{0}$ is in the range from 0 to $f_{rep}$. Since direct photo-detection of optical pulses produces a voltage that only follows their amplitude modulation (heterodyne optical beat frequency), the Fourier spectrum of the MLL pulse train just yields information about difference frequencies in the comb spectrum, i.e., harmonics of $f_{rep}$ (within the photo-detection electrical bandwidth), but not on $f_{ceo}$ which is common to each mode: $f_{n}-f_{m}=n\;f_{rep}+f_{ceo}-m\;f_{rep}-f_{ceo}=\left(n-m\right)f_{rep}$. However, if the comb spectrum covers an optical octave, $f_{ceo}$ can be detected via the so-called f−2f self-referencing scheme. Here, a mode with number *n* in the red wing of the comb, whose frequency is given by $f_{n}=n\;f_{rep}+f_{ceo}$, is frequency-doubled in a nonlinear crystal. Since a mode with number 2n simultaneously oscillates in the blue wing at $f_{2n}=2n\;f_{rep}+f_{ceo}$, the beat note between the frequency-doubled mode and the mode at 2n yields the offset frequency: $2\left(n\;f_{rep}+f_{ceo}\right)-\left(2n\;f_{rep}+f_{ceo}\right)=f_{ceo}$. Such broad optical spectra can be achieved e.g., with continuum generation in highly nonlinear fibers, if the MLL output itself does not have sufficient bandwidth. The first self-referenced frequency combs were obtained with titanium:sapphire (Ti:Sa) lasers (at 800 nm) used in conjunction with photonic crystal fibers for the additional spectral broadening. A few years later, the f−2f self-referencing scheme was also accomplished with an erbium-doped fiber laser (at 1550 nm) spectrally broadened through a highly nonlinear dispersion-flattened fiber, the most commonly used comb system to date [14].

### 2.2. Spectral Coverage

Based on MLL technology, in order to extend OFCs to different wavelengths, significant efforts were directed towards the realization of novel high-pulse-energy diode-pumped lasers (either solid-state or fiber based) along with the corresponding highly nonlinear fibers for continuum generation. In parallel, with regard to spectral regions not addressable directly, nonlinear frequency conversion processes starting from available MLL systems began to be extensively used. Over time, many other effective comb platforms, either MLL-based or not, have also emerged [15,16].

NIR (0.8–2 μm) region—MLL OFCs are commercially available in this range, the most widespread gain media being Ti:Sa and fibres doped with ytterbium [17], erbium [18], thulium or holmium [19]. Yet, new interesting developments, particularly around 1.5 μm wavelength, are taking place to overcome some limitations that characterize the spectrum of frequency combs emitted by MLL systems. Examples include: soliton Kerr combs generated in high-quality-factor micro­resonators, which give access to higher repetition rates (from 10 GHz to 1 THz) [20]; comb architectures based on one or more electro-optic modulators which, in spite of a moderate number of teeth (up to 10,000), exhibit a flat-top spectrum, a freely selectable mode spacing, and a rapidly tunable centre frequency [21].

MIR (2–20 μm) region—In this spectral interval (so called molecular fingerprint region), direct generation of ultra-short pulses, inaugurated by Cr:ZnS/Cr:ZnSe solid-state lasers at 2.4 μm [22], has progressed with a mode-locked Er${}^{3+}$-doped fluoride-glass fiber laser of 200-fs pulse duration at 2.8 μm [23]. Further breakthroughs may also be expected from several MIR gain bands of erbium, thulium and holmium. Remaining on direct OFC sources, remarkable advances have also been achieved with quantum cascade lasers [24,25,26]. Although these can benefit from an ad-hoc tailoring of the spectral emission in the 3–250 micron range, by quantum engineering of the band structure, the extremely short (<1 ps) upper-state lifetime of the intersubband-based gain process strongly hinders passive mode-locking. Nevertheless, under engineered dispersion and gain designs, the QCL multimode operation can be locked into comb modes via a very broadband four-wave-mixing (FWM) parametric generation, enabled by the large third-order ($\chi^{3}$) nonlinearity of the active region. These combs, whose emission profile is not pulsed, cover several spectral bands between 4 and 9 μm at an average power up to the Watt level, with a few hundred optical modes and a typical line spacing in the GHz range. Finally, at wavelengths around 3 μm, where QCLs tend to perform less favorably, the first OFCs based on interband cascade lasers are starting to come out [27,28].

Where direct comb sources are difficult to achieve, mid-infrared OFCs are generated by frequency down-conversion in a nonlinear crystal [29,30]. Concerning difference frequency generation, in the most used approach, the DFG pump and signal beams are derived as two distinct spectral portions from the same broadband NIR OFC [31,32]. Thus, the offset frequency of the generated MIR (idler) comb is inherently zero. This reduces the task of the full comb stabilization to that of the repetition frequency of the initial NIR OFC. As a drawback, an external acousto-optic frequency shifter must be used to displace the idler comb, if needed. In the spectral interval between 2.6 and 5.2 μm, by far the most used crystal is periodically-poled lithium niobate (PPLN), providing (in single-pass configuration) DFG-based MIR combs with power levels in the 100 μW–20 mW interval [33]. Higher power DFG OFCs have also been realized, based either on Raman-induced soliton self-frequency shift in a nonlinear fiber (~240 mW at 2.7–4.2 μm) [34] or on a two-stage optical parametric amplifier (6.7 W average power at 2.9 μm) [35]. Frequency combs at longer MIR wavelengths have been attained with a GaSe crystal (~15 μW at 7.5–12.5 μm, ~4 mW at 8–14 μm) [36,37], with a AgGaS${}_{2}$ crystal (~1.5 mW at 7.5–11.6 μm) [38], and with orientation-patterned (OP) GaP (~60 mW at 6–11 μm) [39]. Compared to DFG-based sources, OPO-based OFCs exhibit higher power levels and offer even wider wavelength tuning ranges [40]. OPO frequency combs have been reported using MgO-PPLN (1.5 W at 2.8–4.8 μm, 30 mW at 2.2–3.7 μm) [41,42], OP-GaAs (70 mW at 2.6–7.5 μm, 10 mW at 5.2–6.2 μm) [43,44], AgGaSe${}_{2}$ (17.5 mW at 4.8–6.0 μm) [45], and OP-GaP (30 mW at 2.3–4.8 μm) [46]. At longer wavelengths, based on a AgGaSe${}_{2}$ crystal, an OPO pumped by a Tm-fiber comb led to the realization of a phase-stabilized OFC continuously tunable from 8.4 to 9.5 μm with a maximum average idler power of 100 mW at 8.5 μm [47]. Effective MIR combs with an instantaneous output spectrum of 3–12.5 μm have also been obtained with subharmonic OPOs based on OP-GaAs as a gain nonlinear crystal, pumped by a Kerr-lens mode-locked 2.35-μm laser [48]. An exceptionally tunable IR frequency comb (1.33–20 μm, 40 mW at 9 μm) was also achieved by combination of DFG and OPO [49]. Lately, interesting results between 0.3 and 11.6 μm are also being obtained with LiGaS${}_{2}$ crystals [50].

THz (0.3–15 THz, 20 μm–1 mm) region—So far, the main approach for generating a THz frequency comb has involved nonlinear frequency down-conversion of a NIR OFC, using photomixing in photoconductive antennas [51] or optical rectification in crystals [52]. This yields an offset-free comb having a maximum average power of a few microwatts and the same line spacing as the starting NIR OFC. A valuable alternative is represented by THz QCLs [53,54] which, based on the aforementioned FWM mechanism, have already demonstrated frequency-comb operation (almost over an octave) around a central frequency of 3 THz (100 μm) with a power of 10 mW and a line spacing of 13 GHz [55]. A THz harmonic frequency comb in the range of 2.2–3.3 THz (mode spacing of 157 GHz, continuous wave power up to 5 μW) was also reported, based on intra-cavity difference-frequency generation from a MIR QCL [56].

### 2.3. OFC Stabilization and Absolute Frequency Metrology Schemes

Starting with MLL OFCs, in the absence of active stabilization, $f_{rep}$ and $f_{ceo}$ would be free to drift or fluctuate due to changes in the cavity length, refractive index of laser optics, and Kerr-type nonlinear effects [57,58,59]. $f_{rep}$ stabilization is commonly implemented by acting on the laser cavity length via a mirror mounted on an intra-cavity piezo-electric transducer. Faster actuation and hence larger stabilization bandwidths (approaching 1 MHz) can be reached using intra-cavity electro-optic modulators (EOMs), both in fiber and solid-state laser systems [60,61,62]. Concerning $f_{ceo}$, the phase error signal is typically fed back to the pump power of the laser, the maximum attainable phase lock bandwidth being set by the gain medium stimulated lifetime. Up-to-ten-times faster actuation methods have also been demonstrated, including loss modulation through intra-cavity graphene electro-optic modulators in fiber lasers [63], and loss modulation of the semiconductor saturable-absorber mirror or gain modulation via stimulated emission in diode-pumped solid-state lasers [64]. Moreover, for comb sources not deriving from MLLs, fluctuations of the two OFC degrees of freedom, coming from the most varied mechanisms, must be suppressed. This is in general accomplished via tight stabilization against suitable frequency standards; in fact, if the latter are linked to the Cs-clock primary standard, the frequency of every optical mode, $f_{n}$, is known absolutely with respect to the definition of the SI second.

For a self-referenced OFC, the most popular approach, so called microwave-to-optical multiplication, consists of phase-locking both $f_{ceo}$ and $f_{rep}$ against a Cs-clock-disciplined MW oscillator, most commonly a GPS-disciplined Rb clock [65] or, in more demanding applications, hydrogen maser [66]. In this case, the phase-noise power spectral density of the reference oscillator is roughly multiplied by the mode number squared ($n^{2}$~$10^{8}\ldots 10^{10}$), thus leading to a comb with optical line widths between a few tens and several hundred kHz. In general, the noise equivalent linewidth of the comb modes is lower the further apart the two locking points are [7]. As a result, the best performance is ensured by optical-to-microwave division, where the locking points are separated by hundreds of THz, with $f_{rep}$ constrained via stabilization of a mode in the optical domain and $f_{ceo}$ phase-locked to a RF reference near 0 Hz (which can also be a subharmonic of $f_{rep}$). Actually, by phase-locking the heterodyne beat note between one of the comb modes and a sub-Hz-linewidth optical frequency source (typically a CW laser locked to an ultra-low-expansion high-finesse cavity) to a Cs clock, ultra-low-noise OFCs with optical line widths in the mHz range can be achieved [67]. Finally, when $f_{ceo}$ is not accessible, full comb stabilization is accomplished by phase-locking $f_{rep}$ to a MW/RF standard and one comb tooth to an optical reference.

Aside from full phase stabilization, electronic phase noise removal from the comb parameters can be implemented. In the most effective approach, the OFC only acts as a transfer oscillator, where the drift of $f_{ceo}$ and $f_{rep}$ is loosely controlled in order to precisely track their electronic signals and correct the noise excursion across the whole comb modes, via a clever combination of signals involving the beat-note with an optical reference [68]. As a result, the output RF signal only contains the down-scaled phase noise of the optical reference without degradation by the OFC.

### 2.4. Direct Frequency Comb Spectroscopy

**Figure 1:**
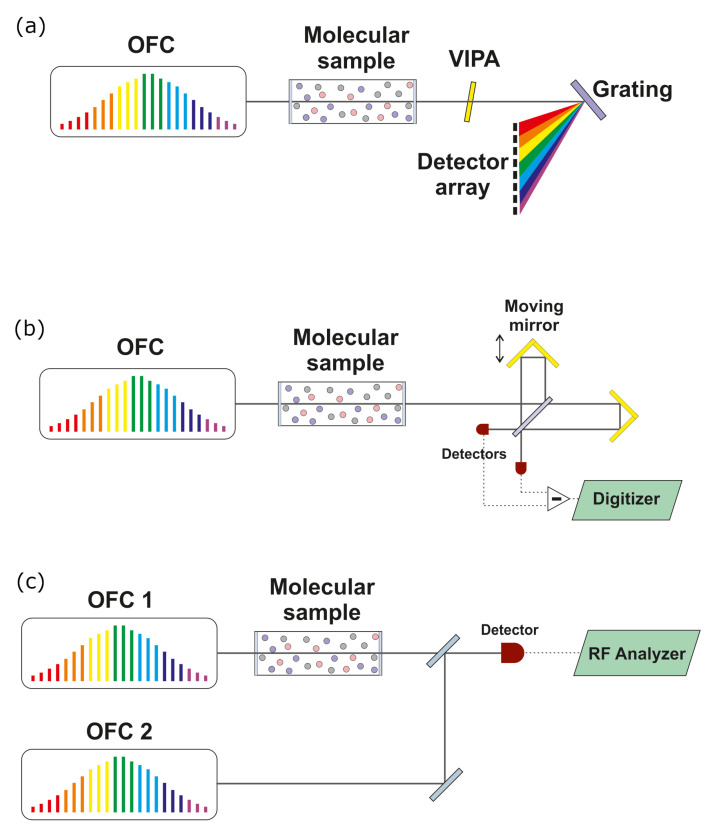
Most used direct frequency comb spectroscopy (DFCS) schemes. (**a**) Frequency comb spectrometry with a disperser for absorption measurements. Here, the optical frequency comb (OFC) light, focused in a line, illuminates the virtual image phased arrays (VIPA) etalon; different VIPA orders are then separated through subsequent dispersion by a classical grating. The final output is detected by a charge-coupled device (CCD) array where each pixel is illuminated by an OFC mode. (**b**) Frequency comb Fourier transform spectroscopy with a scanning Michelson interferometer. (**c**) Dual-comb spectroscopy with two phase-locked OFCs, with one comb interrogating the sample and the other acting as a local oscillator. Here, both the absorption and the dispersion of the sample can be measured. Adapted from [6].

An infrared OFC, fully stabilized and absolutely frequency-referenced according to the most appropriate of the above schemes, is a powerful tool for direct broadband spectroscopy of vibrational and rotational molecular transitions (see Figure 1). Indeed, the huge number of phase-coherent comb components provides a massive set of parallel detection channels to gather high-resolution spectroscopic information [69]. The self-calibration of the frequency scale of the spectra with atomic-clock accuracy, along with the negligible contribution of the instrumental line-shape, enables determinations of all spectral parameters with great accuracy. To take full advantage of the unique features of OFCs for spectroscopy, detection schemes must be capable of measuring the amplitude (or relative phase) variations on individual comb modes after sample interaction. Concerning single direct comb absorption spectroscopy, detection schemes are essentially based on virtual image phased arrays (VIPA) [70,71] or Fourier transform spectrometers [72,73]. An alternative, extremely effective approach, known as dual-comb spectroscopy [74,75,76], makes use of two frequency combs of slightly differing line spacing to down-convert the information imprinted on the comb teeth from the optical domain to the RF domain on a single fast photodetector.

Moreover, the detection sensitivity of DFCS can be greatly improved by using an enhancement cavity, where the molecular sample is placed between high-reflectivity mirrors to substantially increase the absorption path length. In single cavity-enhanced (CE) DFCS, the comb mode spacing is matched with the free spectral range (FSR) of the high-finesse cavity, such that each OFC line is efficiently coupled into a specific longitudinal cavity mode. Then, the cavity transmission is analyzed by a VIPA-grating detector. In a sense, CE-DFCS is virtually equivalent to thousands of simultaneous, highly sensitive, absorption measurements with thousands of narrow linewidth lasers. The maximum cavity acceptance bandwidth (typically less than 100 nm) is limited by dispersion due to the cavity mirrors which introduces a frequency dependence of the FSR. To overcome this drawback and to avoid, at the same time, the use of the VIPA element, the modes of the comb and those of the cavity can be arranged like a Vernier scale [77,78].

**Figure 2:**
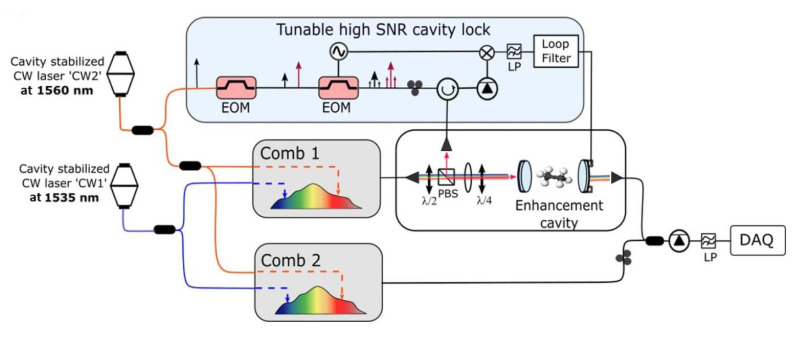
Schematic of dual cavity-enhanced (CE)-direct frequency comb spectroscopy (DFCS). Part of the light from one of the reference lasers is frequency-shifted using an electro-optic modulator (EOM), while a second EOM generates sidebands for Pound-Drever-Hall (PDH) locking of the spectroscopic enhancement cavity to the frequency-shifted reference laser. By changing the modulation frequency of the first EOM, one can align the cavity resonances with the comb modes in any location across the spectrum. Reprinted with permission from [80] ©The Optical Society.

In dual CE-DFCS [79,80], the pair of OFCs are fully stabilized by phase-locking two teeth of each comb to as many cavity-stabilized CW lasers (see Figure 2). After that, the light from comb 1 ($f_{rep,1}$) is coupled to the spectroscopic enhancement cavity where it interacts with the gas sample. The cavity-transmitted light beats with comb 2 ($f_{rep,2}=f_{rep,1}+\delta f$) on a fast detector. The down-converted signal is eventually digitized and processed.

### 2.5. Frequency Comb Assisted Spectroscopy

In FCAS, a tunable, narrow-linewidth CW laser is instead used as the spectroscopic probe source, the OFC only serving as a stable and absolute frequency ruler. When a parametrically generated MIR frequency comb is used, the beat note at frequency $f_{beat}$ between the laser and its closest comb tooth ($\bar{n}$) is phase-locked by an electronic servo to a given local oscillator (linked, in turn, to a Cs clock) by feeding back proper corrections to the actuators (internal and/or external) available for the specific laser system in use (piezoelectric transducer, supply current, temperature, acousto-optic modulator, etc.). In this way, the laser frequency reads

(2) $$f_{laser}=f_{\bar{n}}\pm f_{beat}$$

where $f_{beat}$ at a given time is provided by a precision frequency counter; if $f_{beat}$ is observed to increase (decrease) when $f_{rep}$ is increased very slightly, then the negative (positive) sign must be used. After that, $f_{laser}$ can be scanned across the molecular transition by tuning either $f_{\bar{n}}$ (typically via $f_{rep}$) or $f_{beat}$. Coherently locking a CW laser to an OFC by the servo feedback phase-locked loop (PLL) method works very well with extended cavity diode lasers (ECDLs), where wide locking bandwidths, even beyond the MHz, can be achieved by direct feedback control of the injection current of the diode laser. Conversely, in most solid-state and fiber lasers, the pump-gain process usually limits the locking bandwidth to a very few hundred kHz. In such cases, a very effective solution is represented by a feed-forward method, based on an acousto-optic frequency shifter (AOFS) without the PLL, originally demonstrated for $f_{ceo}$ stabilization. Here, the beat note signal detected upstream between the CW laser and the nearest comb mode, $f_{beat}\left(t\right)=f_{laser}-f_{\bar{n}}$ directly drives the AOFS ($f_{AOFS}\left(t\right)=-f_{beat}$), so that the frequency of the first-order diffracted beam matches exactly the comb mode frequency: $f_{1st}=f_{laser}+f_{AOFS}\left(t\right)=f_{laser}-f_{beat}=f_{\bar{n}}$. In this way, a maximum control bandwidth around the MHz can be reached, only limited by the transit time of the acoustic wave inside the AOFS [81].

Aside from direct referencing to a parametrically generated IR frequency comb, the CW spectroscopic probe laser can be frequency up-converted via second harmonic generation (SHG) or sum-frequency generation (SFG) in a nonlinear crystal and then referenced to a NIR OFC. Finally, when using a CW DFG or OPO laser source for the spectroscopic interrogation, a valuable option consists of referencing pump and signal wavelengths to a NIR OFC [82].

## 3. Buffer Gas Cooling

With the exception of very few special cases, the powerful laser cooling techniques so far developed to chill and confine atoms are not straightforward to apply to molecules, due the lack of a closed set of cycling transitions (primarily related to the absence of strict selection rules between vibrational levels). Indirect cooling methods for molecules associate pairs of ultra-low-temperature alkali-metal atoms through laser or magnetic assisted processes to form an usually diatomic molecule at very low translational temperature, but in an excited state; then, coherent transfer into the ro-vibrational ground state creates a quantum degenerate molecular gas [83]. The main drawback of this approach is that it only works for molecules whose constituent atoms can be laser cooled and trapped, thus excluding many chemical species that are relevant for spectroscopic studies (hydrides, nitrides, oxides, fluorides, etc.). Meanwhile, a number of direct techniques has been demonstrated to bring stable molecules into the cold temperature regime, encompassing Stark [84] or Zeeman deceleration [85], electro-optical Sisyphus cooling [86], cryofuges [87], electromagnetic traps [88] and buffer gas cooling.

BGC is by far the most universal technique to produce cold molecular samples, as it does not depend on properties like internal energy structure or electric dipole moment, but just works by thermalizing, via elastic collisions, the species of interest with a cryogenic bath of inert (buffer) gas atoms, such as helium or neon [89,90]. The minimum attainable temperature corresponds to that for which the buffer gas has an appreciable vapor pressure (~4 K for ${}^{4}$He, ~300 mK for ${}^{3}$He). This dramatically reduces the number of populated (internal) rotational and vibrational states, beneficially influencing a variety of research domains ranging from high-resolution molecular spectroscopy, to precise control of chemical reactions, to study of novel dynamics in low-energy collisions. Within the last two decades, BGC has been applied to a number of species including small, light and chemically stable polyatomic molecules (e.g., NH${}_{3}$, CH${}_{3}$F, CF${}_{3}$H, C${}_{2}$H${}_{2}$), radicals (e.g., NH, CaF, SrF), heavy species (e.g., ThO [91], YbF [92], YbOH [93], TlF [94], BaH [95], BaF [96]), as well as big biomolecules like trans-cinnamaldehyde or gauche-isoprene [97,98].

Besides, starting from molecular beams emerging from cryogenic buffer gas sources, laser cooling has recently demonstrated for both diatomic and small polyatomic molecules [99], leading to a series of groundbreaking experiments with trapped samples of SrF [100], CaF [101], and YO [102,103] at low μK temperatures.

### 3.1. Typical Experimental Setup and Collision Mechanism

The collisional cooling process takes place inside a cell (commonly a hollow copper parallelepiped or cylinder of length $L_{cell}$ and cross sectional area $A_{cell}$, for a volume $V_{cell}$ of several cm${}^{3}$) that is held at a fixed temperature $T_{cell}$ by a cryogenic refrigerator (a pulse-tube cryocooler or a liquid-He–liquid-N${}_{2}$ dewar), typically between 1 and 20 K. Buffer gas, whose flux is precisely controlled by means of a mass flow controller, is let into the cell at a cryogenic temperature $T_{b}$ (close to $T_{cell}$) by a pipeline thermally anchored to the various cold stages of the cryostat. An aperture (or nozzle) with a characteristic area $A_{ap}$ of several mm${}^{2}$ on the opposite side of the cell lets the buffer gas spray out as a beam. Thus, after a short transient (a few tens of ms), stationary gas densities (gas pressures) will be established inside the buffer cell. The steady-state density $n_{0,b}$ is found by equating the flow rate out of the cell, $f_{out}=\left(A_{ap}\;{\bar{v}}_{0,b}\;n_{0,b}\right)/4$, with the input flow, $f_{0,b}$:

(3) $$n_{0,b}=\frac{4f_{0,b}}{A_{ap}\;{\bar{v}}_{0,b}}$$

where

(4) $${\bar{v}}_{0,b}=\sqrt{\frac{8k_{B}T_{b}}{\pi m_{b}}}$$

is the mean thermal velocity of the gas atoms of mass $m_{b}$. With typical operational parameters, a flow of 1 SCCM (standard cubic centimeters per minute) corresponds to $n_{0,b}\sim 10^{15}$ cm${}^{-3}$. Typically, the $n_{0,b}$ value is kept below $10^{17}$ cm${}^{-3}$, which is low enough to prevent three-body-collision cluster formation involving the target molecule yet high enough to guarantee the number of collisions necessary for thermalization before the molecules escape the cell.

**Figure 3:**
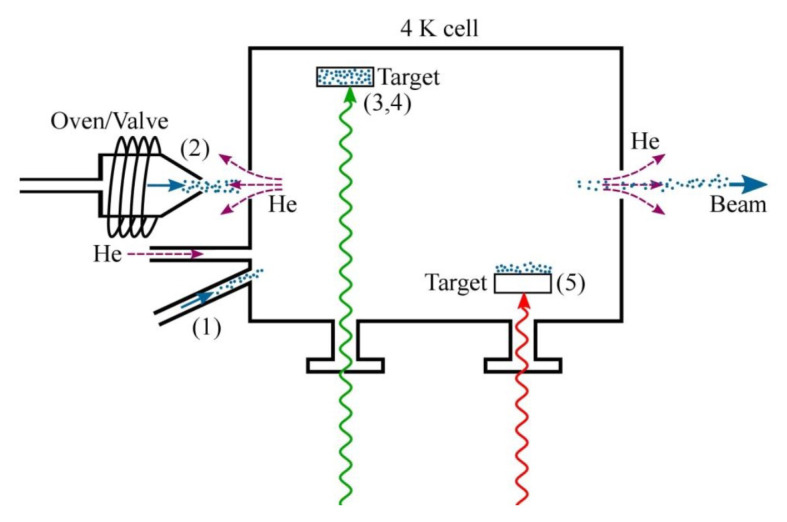
Different molecule-loading mechanisms into a buffer gas cell: (1) Capillary filling, (2) loading from a valve or oven, (3) direct laser ablation of the target, (4) matrix-assisted desorption, (5) laser-induced acoustic desorption of a solid target containing the molecules of interest [105]. Reproduced with permission from [104].

As shown in Figure 3, different loading mechanisms can be adopted for the molecular species of interest [104]. If the latter has an appreciable vapor pressure at convenient temperatures, then one can simply flow it into the cell through a gas fill line, just like the buffer gas. In this case, however, the pipeline must be stabilized to a temperature which prevents freezing of molecules inside, as well as the inlet must be thermally isolated from the cell. To this aim, one or more thermometers and heaters, inserted in a proportional-integral-derivative (PID) feedback loop, are placed along the pipeline. The primary advantage of the capillary filling method is that it can be used to create high-flux beams that are continuous. Conversely, for species that require unreasonable temperatures (1000 K) to obtain an appreciable vapor pressure, laser ablation is the main option. Here, a high-energy pulsed laser (typically a Nd:YAG delivering several hundred mJ at 1064 nm in a few nanoseconds) is focused onto a solid precursor target located inside the buffer gas cell. Quite often, the desired molecular species has a stable solid phase (glass or ceramic in the case of BaF, CaH, SrF, YO, ThO, and others) which can be used as a precursor in the ablation process. Known chemical reactions can also be exploited to realize ablation targets that mix multiple species; for example, a heated mixture of SrF${}_{2}$+B can be used to produce SrF. The main drawback is that ablation is a strongly nonthermal process which can even lead to the formation of plasmas and complex plumes with temperatures of several thousand kelvins. These problems can be mitigated by carefully fixing the various operational (pulse energy and duration, and focus of the ablation laser) and geometric parameters (cell design, target location), allowing proper thermalization of the species.

Both in capillary filling and laser ablation, the species behaves only as a trace component, its density being typically <1% of that of the buffer gas which, therefore, alone determines the gas flow properties. Once injected into the buffer gas cell at a temperature $T_{s}\left(0\right)$, the molecules undergo random collisions with the cold atoms, progressively transferring energy to the thermal bath until they reach, after $N_{coll}$ collisions, a temperature $T_{s}\left(N_{coll}\right)$ close to $T_{b}$. Assuming the hard-sphere model, one finds

(5) $$T_{s}\left(N_{coll}\right)≃T_{b}+T_{s}\left(0\right)\;e^{-N_{coll}/k}$$

where

(6) $$k=\frac{{\left(m_{b}+m_{s}\right)}^{2}}{2\;m_{b}\;m_{s}}$$

with $m_{s}$ being the molecular mass. For a reasonable system, the number of collisions for a complete thermalization does not exceed 100. Then, denoting with $\sigma_{s-b}$ the thermally-averaged elastic collision cross section, the mean free path is given by

(7) $$\lambda_{s-b}=\frac{1}{n_{0,b}\;\sigma_{s-b}{\left(1+m_{s}/m_{b}\right)}^{1/2}}$$

which corresponds to a thermalization length $N_{coll}\cdot \lambda_{s-b}$ lower than 1 cm, for usual $\sigma_{s-b}$ and $n_{0,b}$ values. Instead, if the species is loaded via ablation, then the buffer gas will itself be heated and require some time to cool back down to $T_{cell}$; as a consequence, the time required for the species to reach the steady-state cell temperature can go up to several milliseconds.

The described cooling mechanism only pertains to the translational temperature (related to the mean square velocity), with which rotational, vibrational and electronic temperatures (related to the Boltzmann distribution of corresponding internal states) coincide only at equilibrium. Rotational relaxation cross sections for molecules with helium buffer gas are in the range $\sigma_{rot}=10^{-15}-10^{-16}$ cm${}^{2}$, which increases the thermalization length by 1 or 2 orders of magnitude; as a result, rotational thermalization requires cell lengths of a few centimeters. Cross sections for vibrational relaxation are even smaller [106,107]; however, in many cases (for example when using capillary filling), the injected molecules are already in vibrational fundamental state.

Once the molecules are thermalized, one has to minimize the probability of them sticking to the cell walls. This will maximize the percentage of molecules able to escape through the exit aperture, hence contributing to the formation of the beam. For this purpose, the diffusion timescale of the molecules to the cell

(8) $$\tau_{diff}=\frac{16}{9\pi }\;\frac{A_{cell}^{2}\;n_{0,b}\;\sigma_{s-b}}{{\bar{v}}_{0,b}}$$

must be longer than the extraction or pump-out timescale of the species from the cell (which can be safely approximated to the pump-out timescale of the buffer gas atoms)

(9) $$\tau_{pump}=\frac{4V_{cell}}{A_{ap}\;{\bar{v}}_{0,b}}$$

Typically, both $\tau_{diff}$ and $\tau_{pump}$ are in the range 1–10 ms. At this point, if the ratio $\gamma_{cell}=\tau_{diff}/\tau_{pump}$ exceeds 1, then the molecules are mostly extracted from the cell before sticking to the walls. The corresponding overall extraction efficiency can reach a few tens of percent, resulting in a molecular beam of increased brightness. In this regime, so called hydrodynamic entrainment, the molecular beam is characterized by a velocity distribution that can vary considerably from that inside the cell. Unfortunately, the functional dependence of the molecular flux on $\gamma_{cell}$ is not yet clear, and varies significantly from species to species even with the same experimental setup [9,108].

### 3.2. Molecular Beam Generation and Properties

Eventually, a molecular beam is formed by letting the gas pass from the buffer cell into an evacuated chamber. As a first precaution, in order to prevent the species of interest from sticking to the sides of the nozzle, this latter should be thin-walled (typically < 0.5 mm) [10]. After that, the following aspects related to the vacuum pumping system must be carefully considered. Since the blackbody heat load on a 4-K surface from a 300-K environment is about 50 mW per square centimeter, in order to avoid overwhelming of the cryocooler, it is necessary to surround the buffer cell with cold surfaces, or radiation shields. These are thermally conductive boxes (typically made of copper panels), cooled by the different stages of the cryostat. However, enclosing the cell in radiation shields strongly reduces the gas conductance to the external vacuum pumps, which would cause the residual pressure to grow to the point of hindering the beam formation. This drawback can be overcome by covering the inside of the 4-K radiation shield with activated charcoal which, when cooled below 10 K, becomes a cryopump for the buffer gas with a pumping speed of several liters per second per square centimeter. As the charcoal fills up with the buffer gas, typically after a few hours, the pumping speed drops considerably, which ends up degrading the beam properties; at that point, it is necessary to warm up the charcoal (several tens of Kelvin) and pump out the desorbed buffer gas with a turbo-molecular pump.

A primary parameter governing the beam properties is the Reynolds number, which can be expressed as twice the number of collisions near the aperture:

(10) $$Re≃2\;d_{ap}/\lambda_{b-b}$$

where $d_{ap}$ is the aperture diameter. Accordingly, the following three flow regimes can be roughly identified.

- Effusive regime, Re<1: there are typically no collisions near the aperture, such that the beam properties are essentially a sampling of the thermal distribution inside in the cell.
- Partially hydrodynamic regime, 1≤Re≤100: collisions near the aperture increase significantly in number, essentially in the forward direction (due to geometric constraints). Since the atomic velocity is much larger than the molecular one, the net effect is a boosting of the molecules in the forward direction. While generating a molecular beam with substantially different characteristics with respect to the thermal distribution inside the cell, the number of collisions is not yet so high as to make the outgoing flow fluid-like. Buffer gas beams typically operate in this regime, which is able to produce slower beams that contain more molecules.
- Supersonic or fully hydrodynamic regime, Re>100: the buffer or carrier gas begins to behave more like a fluid. The molecules of interest are entrained in the carrier gas which cools by adiabatic expansion from a high-pressure region into a vacuum. The initial thermal energy of the carrier gas is converted to forward kinetic energy, resulting in a fast-moving molecular beam.

Focusing here on the partially hydrodynamic regime, the mean forward (longitudinal) velocity of the molecules in the beam can be expressed as

(11) $${\bar{v}}_{\|,s}≃\left\{\begin{array}{cc} 1.2\;{\bar{v}}_{0,s}+0.6\;{\bar{v}}_{0,b}\;Re\;\frac{m_{b}}{m_{s}} & 1<Re<10\\ 1.4\;{\bar{v}}_{0,b}\;\sqrt{1-4Re^{-4/5}} & 10\leq Re<100 \end{array}\right.$$

where

(12) $${\bar{v}}_{0,s}=\sqrt{\frac{8k_{B}T_{b}}{\pi m_{s}}}$$

is the mean thermal velocity of the molecules inside the cell. Obviously, the mean forward velocity approaches that of a supersonic beam ($1.4\;{\bar{v}}_{0,b}$) for large Re values, or that of an effusive beam ($1.2\;{\bar{v}}_{0,s}$) for small Re values (typically, $m_{s}\gg m_{b}$). For sufficiently high Re values, the forward velocity spread, $\Delta {\bar{v}}_{\|,s}$, can begin to decrease due to isentropic expansion of the buffer gas into the vacuum region, causing the translational temperature in the longitudinal direction to drop below the cell temperature.

Concerning the transverse velocity spread, $\Delta v_{⊥,s}$, it is more complicated to model. However, it should increase linearly (above some Re) with increasing Re. Finally, as Re is further increased (around 100), it saturates at the value $\Delta v_{⊥,s}≃1.5\;{\bar{v}}_{0,s}$. Then, the full angular divergence of the beam will begin to decrease

(13) $$\Delta \theta =2\arctan\frac{\Delta v_{⊥,s}/2}{{\bar{v}}_{\|,s}}≃2\;\sqrt{\frac{m_{b}}{m_{s}}}$$

Notice that this can be much smaller than the corresponding divergence 2π/3 for an effusive beam or 1.4 for a supersonic beam. Since experiments with molecular beams are often performed at a distance from the cell aperture that is many times larger than $d_{ap}$, this smaller angular divergence contributes to the typically large brightness of buffer gas beams. Skimmers may also be used to further collimate a buffer-gas-cooled beam and create differentially pumped regions.

Being characterized by greater brilliance, slower laboratory-frame velocities, and the option of continuous-mode operation (without the need for significant pumping infrastructure), over the time, buffer-gas-cooled beams have become increasingly popular at the expense of traditional supersonic jets. Furthermore, on the basis of detailed simulations (finite-element flow-field or Monte Carlo trajectory), buffer-gas cell design (cell shape, position of gas inlets, nozzle shape, etc.) is constantly improved, leading to buffer-gas-cooled beams with even higher fluxes and lower speeds [109,110]. The typical brightness of a buffer-gas-cooled molecular beam is in the range $10^{8}-10^{12}$ s${}^{-1}$ and $10^{8}-10^{12}$ steradian${}^{-1}$ pulse${}^{-1}$ for continuous and pulsed mode, respectively; ${\bar{v}}_{\|,s}$ values from 40 to 200 m/s and $\Delta {\bar{v}}_{\|,s}$ as low as 25 m/s have been observed.

## 4. Cavity-Enhanced Frequency Comb Spectroscopy of Buffer-Gas-Cooled Molecules

Despite the wide availability of infrared comb systems and buffer-gas-cooled molecular species, OFC-based ro-vibrational spectroscopic studies of cold ground-state molecules are still very few.

### 4.1. DFCS of Complex Molecules

Broadband precision spectroscopy studies in the MIR are typically limited to small, simple molecules in order to avoid spectral congestion. Indeed, every additional atom considerably increases the number of ro-vibrational states populated at a given internal temperature. Moreover, when excited by an IR photon, these ro-vibrational states may be strongly coupled to a crowded manifold of background dark states, leading to intramolecular vibrational redistribution. This spectral congestion is exacerbated by the Doppler broadening of transitions, due to the finite translational temperature. By cooling molecular translational and rotational temperatures, BGC greatly mitigates these problems, enabling quantum state–resolved rovibrational spectroscopy even for large and heavy molecular systems.

**Figure 4:**
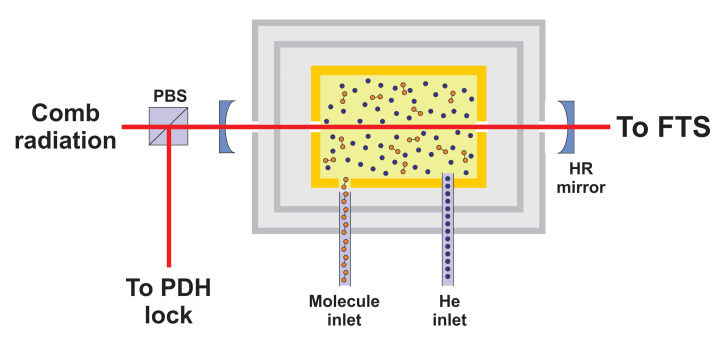
Simplified view of the apparatus used for CE-DFCS of buffer-gas-cooled gas-phase complex molecules. MIR frequency comb light is coupled to a high-finesse (≃6000 both at 3 and 8.5 μm wavelength) cavity surrounding the 10-K buffer gas cell. Comb light reflected from the cavity is used to generate a Pound–Drever–Hall (PDH) error signal to lock the comb to the cavity. The PDH lock guarantees efficient on-resonance cavity transmission over a simultaneous transmission bandwidth of 100 nm (limited by dispersion of the HR mirrors); the spectrum of the transmitted comb light is read out by a Fourier-transform (FT) spectrometer or a VIPA. By adjusting the cavity free-spectral-range (FSR), an 8:5 or 2:1 $f_{rep}$-to-FSR ratio can be selected. Comb modes not resonant with the cavity are rejected, resulting in a sparser transmitted comb spectrum, which facilitates single comb mode resolution with the FTS or VIPA. Adapted from [111].

As shown in Figure 4, combination of BGC and CE-DFCS has indeed succeeded in recording significantly improved spectra (compared to room-temperature studies) of several organic molecules of fundamental and astrochemical relevance, such as vinyl bromide (CH${}_{2}$CHBr), adamantane (C${}_{10}$H${}_{16}$), diamantane (C${}_{14}$H${}_{20}$), nitromethane (CH${}_{3}$NO${}_{2}$), naphthalene (C${}_{10}$H${}_{8}$), hexamethylenetetramine (C${}_{6}$H${}_{12}$N${}_{4}$) in the C–H stretching fundamental region around 3-μm wavelength [111,112]. Here, capillary filling is used for the species of interest, and the frequency comb light (100 mW at 3.0–3.3 μm) comes from an Yb fiber-pumped OPO. A similar setup has been used for spectroscopy of fullerene (C${}_{60}$) [113]. In this case, a 950-K copper oven sublimates solid C${}_{60}$ samples, generating gas-phase molecules with an average internal energy of 6 to 8 eV per molecule populating from 1026 to 1030 vibrational quantum states. OFC radiation, centered near 8.5 μm, is produced via DFG in an OP GaP crystal, starting from two NIR OFCs originating from a single mode-locked Er-doped fiber laser. In both the experiments, the recorded absorption spectra point out the significant gains associated with cooling the species down to 10 K: molecules have much narrower Doppler-broadened linewidths and occupy many fewer and lower rovibrational energy levels, leading to distinguishable absorption lines with enhanced peak amplitudes.

### 4.2. FCAS of Acetylene

BGC offers great opportunities for improving the ultimate attainable precision in FCAS of simple molecules, too. In this context, significant results have been obtained with buffer-gas-cooled acetylene (C${}_{2}$H${}_{2}$), both in the form of a beam and inside the cryogenic cell.

Acetylene has always been the subject of extensive spectroscopic studies. First, the paradigmatic carbon–carbon triple bond is crucial for understanding fundamental quantum chemistry processes in molecular beams (reactions and formation of van der Waals complexes). Moreover, much work in the field of high-resolution spectroscopy has been motivated by the demand for improved frequency standards in the telecommunications range. Trace-molecule spectroscopy of acetylene is of considerable interest also in atmospheric chemistry in connection with pollution control. Finally, acetylene is formed, by photolysis of methane, in the atmospheres of giant planets as well as in various other stellar and interstellar environments, thus playing a key role in astrophysics and astrobiology.

**Figure 5:**
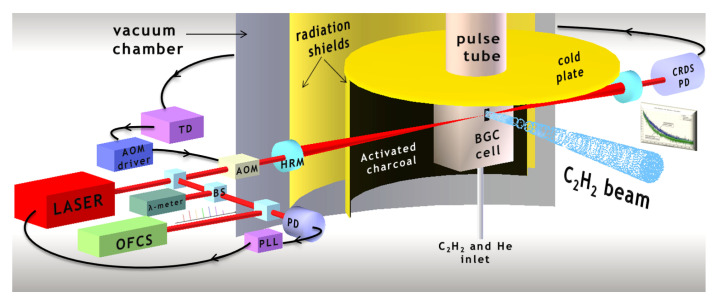
Layout of the experimental setup for OFC-assited cavity ring-down spectroscopy (CRDS) of a 10-K beam of acetylene, consisting of two main blocks: the buffer-gas-cooling (BGC) source and the laser spectromter. CRDS signals, measured at a distance of 1 cm from the BGC cell exit, exhibit signal-to-noise ratios exceeding 200, for injection fluxes of 8 (helium) and 4 (acetylene) SCCM (standard cubic centimeters per minute). Reprinted with permission from [114] *©* The Royal Society of Chemistry.

In a first experiment, as shown in Figure 5, cavity ring-down spectroscopy (CRDS) of a 10-K beam was performed using the $g\to (\nu_{1}+\nu_{3})$ R(1) ro-vibrational transition at 1.5-μm wavelength [114]. With regard to the spectrometer, the laser source is a continuous-wave (CW) external-cavity diode laser (ECDL) tunable between 1470 and 1570 nm, with a maximum output power of 30 mW and a free-running emission linewidth less than 50 kHz (at 5 ms). One portion of the main output beam is beaten against the *N*th tooth of an OFCS (MenloSystems, FC-1500-250-WG), providing a note at frequency $f_{beat}$. This is phase-locked by an electronic servo to a local oscillator by feeding back corrections to the piezo transducer of the ECDL. The second portion is passed through a fiber acousto-optic modulator (AOM) whose first-diffracted order is injected into the high-finesse cavity. In this way, the laser emission frequency is reads as

(14) $$f_{laser}=f_{ceo}+N\cdot f_{rep}+f_{beat}+f_{AOM}$$

where $f_{AOM}=80$ MHz is the frequency of the signal driving the AOM. The link to the Cs-clock standard is established by stabilizing both $f_{ceo}$ and $f_{rep}$ against a Rb/GPS-clock-disciplined quartz oscillator; the same reference chain locks the time base of the synthesizers generating the signals at $f_{LO}$ and $f_{AOM}$. Such a frequency chain ensures an accuracy of $10^{-13}$ and a fractional stability (Allan deviation) between $4\times 10^{-13}$ and $8\times 10^{-13}$ for an integration time between 10 and 1000 s. Finally, the integer *N* is determined by measuring the laser frequency with a 0.2 ppm-accuracy wavelength meter. Then, for each $f_{laser}$ value, the molecular absorption is recorded within a CW CRDS scheme. Here, the laser beam is coupled to an high-finesse optical cavity, consisting of two facing high-reflectivity (99.995%) spherical mirrors (3 m radius of curvature, 1 inch diameter) surrounding the buffer gas cell. As a resonance builds up, under a continuous dithering of the optical resonator length, a threshold detector triggers an abrupt switch-off of the AOM. The subsequent ring-down event is acquired by an InGaAs photodetector (5 MHz electrical bandwidth). The average of ≃50 acquisitions is then used to extract the cavity decay time, τ by means of a least-squares fitting routine. The molecular absorption coefficient is eventually recovered as

(15) $$\alpha \left(f_{laser}\right)=\frac{1}{c}\left[\frac{1}{\tau (f_{laser})}-\frac{1}{\tau_{e}}\right]\frac{D}{d}$$

where *D* is the resonator length, *d* the sample length along the laser propagation direction, $\tau_{e}≃45$ μs the empty-cavity decay constant, and *c* the speed of light. Then, fitting the observed absorption profile with a Gaussian distribution, the absolute center frequency of the investigated transition is extracted with an uncertainty of 300 kHz ($1.5\cdot 10^{-9}$ in fractional terms). The full width at half maximum (FWHM) of the Gaussian distribution is also determined (110±1 MHz), corresponding to a molecular temperature of (16.1±0.2) K.

**Figure 6:**
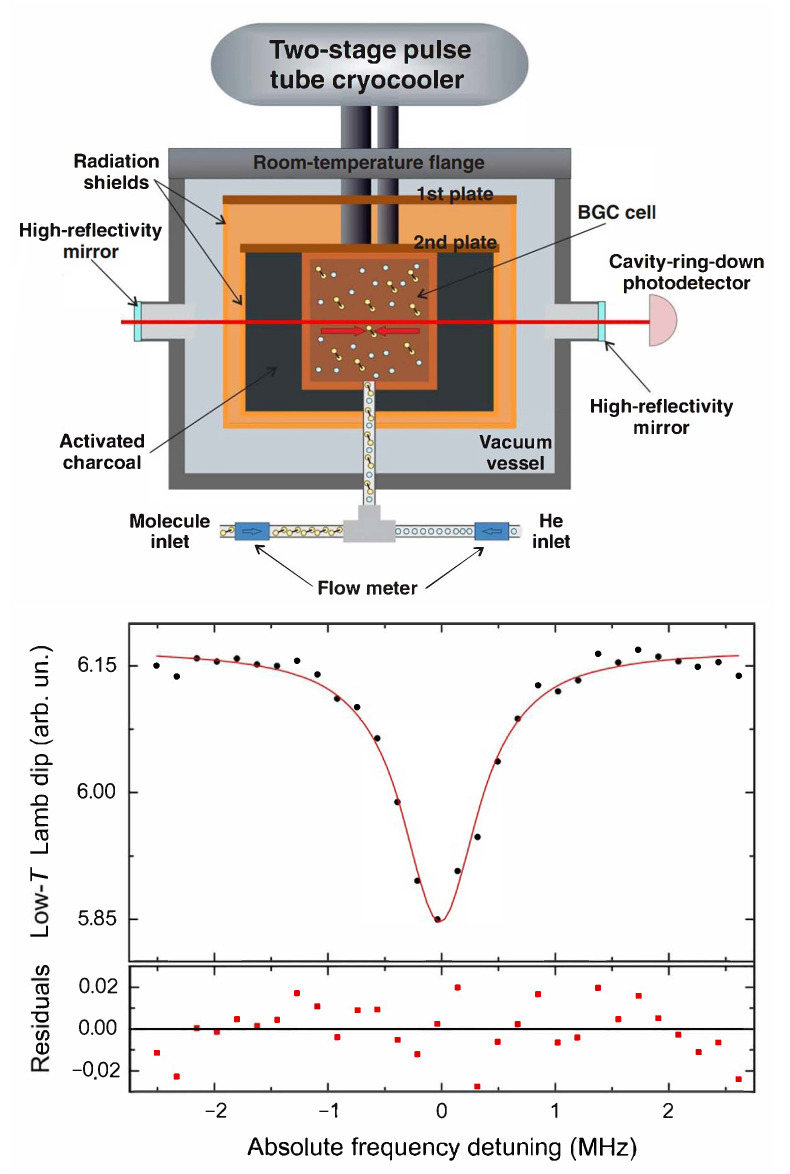
(upper) OFC-assited saturated-absorption cavity ring-down (SCAR) spectroscopy setup for Lamb-dip ro-vibrational measurements on buffer-gas-cooled molecular samples. (lower) Lamb-dip line shape corresponding to a C${}_{2}$H${}_{2}$ sample of ≃20 K, exhibiting a saturation contrast around 8% and a signal-to-noise ratio around 30, obtained for injection fluxes of 6 (helium) and 6 (acetylene) SCCM. Reprinted with permission from [115] *©* The Optical Society.

In a second experiment, the same OFC-referenced probe laser was used to carry out saturated-absorption cavity ring-down (SCAR) spectroscopy of the molecular sample inside the buffer gas cell, demonstrating a general approach to Lamb-dip ro-vibrational spectroscopy of cold stable molecules [115]. In this case, the buffer cell is equipped with two opposite circular holes (5 mm diameter), which are aligned along the axis of the spectroscopic enhancement cavity; this latter is equipped with specially-developed mirror mounts in order to ensure an higher degree of mechanical stability (upper frame of Figure 6). Lamb-dip signals corresponding to the $\left(\nu_{1}+\nu_{3}\right)$ R(1) rovibrational line are then recorded by varying the flux into the cell of either the buffer gas or the molecules. By fitting the obtained sub-Doppler profiles with a Lorentzian line shape, the line-center frequency as well as of the self and foreign collisional broadening coefficients are absolutely determined (lower frame of Figure 6). The best statistical uncertainty on the line-center frequency is 12 kHz ($6\times 10^{-11}$ in fractional terms), with a the full width at half-maximum (FWHM) of ≃800 kHz, mainly ascribable to foreign (i.e., due to the buffer gas) collisional broadening. The overall systematic uncertainty associated with the absolute determination of the center frequency of the selected ro-vibrational line was also estimated: 0.5 kHz. This approach opens the low-temperature regime to accurate measurements of basic spectroscopic parameters for a vast range of gas-phase molecular species. In order to suppress collisional broadening effects and enter the transit-time-limited regime, the next step is to significantly decrease the gas flows entering the buffer cell, while simultaneously improving the spectrometer detection sensitivity. For this purpose, a new mechanical design for the enhancement cavity is under construction, which is able to effectively break off detrimental vibrations from the pulse-tube cryo-cooler. In turn, this will enable the implementation of a high-bandwidth PDH scheme to lock the probe laser to the high-finesse cavity, thus substantially increasing the acquisition rate of ring-down events. Moreover, acquiring the SCAR signal with a 24-bit-vertical-resolution oscilloscope will enable the use of a more comprehensive fitting function to describe the time-dependent saturation level of the absorbing gas [116]; this will make it possible to reject most fluctuations in the empty-cavity decay rate.

In the end, it is worth mentioning an experiment which applies precision FCAS to a large buffer-gas-cooled species, CH${}_{3}$ReO${}_{3}$ (methyltrioxorhenium or MTO), closely related to chiral molecules where parity-violating energy differences between enantiomers are expected to be measurable [117]. Currently, the MTO molecules are produced with a rotational temperature around 6 K by laser ablation inside a cryogenic helium buffer gas cell, and a frequency resolution of 8 MHz (accuracy of 30 MHz) is achieved for the 10.2 μm antisymmetric Re=O stretching mode via QCL-based absorption spectroscopy.

## 5. Conclusions and Perspectives

In conclusion, applying OFC-based spectroscopic interrogation schemes to buffer-gas-cooled samples offers unique opportunities to harness the numerous but complex molecular degrees of freedom (internal structure and strong intermolecular interactions), in view of novel IR precision measurements.

One one hand, DFCS is an invaluable approach for studying very large species, enabling complex molecules and their kinetics to be studied with orders-of-magnitude improvements in efficiency, spectral resolution and specificity. In this respect, interesting developments may arise from the application of dual CE-DFCS to buffer-gas-cooled molecules.

On the other hand, as for metrology applications, new ultra-precise frequency measurements on cold molecules may be accomplished by the implementation of more sophisticated FCAS schemes. In addition to the aforementioned SCAR approach, other effective CE spectroscopic techniques can be used, ranging from cavity-enhanced optical-heterodyne molecular spectroscopy (NICE-OHMS) [118,119,120] to optical feedback frequency-stabilized cavity ring-down spectroscopy (OFFS-CRDS) [121]. Furthermore, to greatly increase the spectroscopic interrogation time, which sets the ultimate achievable resolution in the center frequency measurement of the molecular transition, the favorable properties of buffer-gas-cooled beams (high brilliance and slow laboratory-frame velocity) can be exploited for the observation of two-zone Ramsey fringes assisted by CE two-photon excitation in the optical domain [122]. In this configuration, fractional accuracy levels below $10^{-14}$ are potentially within the reach. Correspondingly, as for the metrological scheme, the OFC to which the spectroscopic probe laser is phase-locked must be stabilized against a frequency standard with superior performance, such as a Cs primary fountain clock or a state-of-the-art optical frequency standard, as operated by Metrological Institutes and delivered by actively stabilized fiber links [123,124,125,126].

Based on the mix of all these cutting-edge technologies, IR precision laser spectroscopy of buffer-gas-cooled samples is expected to give life, in the near future, to unprecedented-accuracy molecular tests of fundamental physics at the electron volt energy scale [127]. These include the search for hypothetical long-range fifth forces between hadrons [128] or time-reversal violation (electron’s electric dipole moment) [129,130,131,132], detection of parity violation in chiral molecules [104], testing the time stability of the proton-to-electron mass ratio [133,134], as well as precision tests of Quantum Electro-Dynamics [135,136].

## Data Availability

Not applicable.
